# Relationships Between Neurodegeneration and Vascular Damage in Diabetic Retinopathy

**DOI:** 10.3389/fnins.2019.01172

**Published:** 2019-11-08

**Authors:** Maria Grazia Rossino, Massimo Dal Monte, Giovanni Casini

**Affiliations:** ^1^Department of Biology, University of Pisa, Pisa, Italy; ^2^Interdepartmental Research Center Nutrafood “Nutraceuticals and Food for Health”, University of Pisa, Pisa, Italy

**Keywords:** nutraceuticals, antioxidants, neuropeptides, vascular endothelial growth factor, blood-retina barrier

## Abstract

Diabetic retinopathy (DR) is a common complication of diabetes and constitutes a major cause of vision impairment and blindness in the world. DR has long been described exclusively as a microvascular disease of the eye. However, in recent years, a growing interest has been focused on the contribution of neuroretinal degeneration to the pathogenesis of the disease, and there are observations suggesting that neuronal death in the early phases of DR may favor the development of microvascular abnormalities, followed by the full manifestation of the disease. However, the mediators that are involved in the crosslink between neurodegeneration and vascular changes have not yet been identified. According to our hypothesis, vascular endothelial growth factor (VEGF) could probably be the most important connecting link between the death of retinal neurons and the occurrence of microvascular lesions. Indeed, VEGF is known to play important neuroprotective actions; therefore, in the early phases of DR, it may be released in response to neuronal suffering, and it would act as a double-edged weapon inducing both neuroprotective and vasoactive effects. If this hypothesis is correct, then any retinal stress causing neuronal damage should be accompanied by VEGF upregulation and by vascular changes. Similarly, any compound with neuroprotective properties should also induce VEGF downregulation and amelioration of the vascular lesions. In this review, we searched for a correlation between neurodegeneration and vasculopathy in animal models of retinal diseases, examining the effects of different neuroprotective substances, ranging from nutraceuticals to antioxidants to neuropeptides and others and showing that reducing neuronal suffering also prevents overexpression of VEGF and vascular complications. Taken together, the reviewed evidence highlights the crucial role played by mediators such as VEGF in the relationship between retinal neuronal damage and vascular alterations and suggests that the use of neuroprotective substances could be an efficient strategy to prevent the onset or to retard the development of DR.

## Introduction

Diabetes is a disease affecting a growing number of people worldwide. It is expected to increase to a little <700 million by 2045, with almost half of diabetics suffering from the slowly progressive type 2 diabetes, which in many cases remains undiagnosed (Cho et al., 2018). Type 2 diabetes is the main cause of diabetes in the population aged 40–74 years, although there is an increasing number of people aged <40 suffering from this form of the disease (Pantalone et al., 2015). Untreated or poorly controlled diabetes may lead to the appearance of serious complications, including diabetic retinopathy (DR). DR is the most common complication of diabetes and the leading cause of preventable visual impairment in the working age population in developed countries. It is also one of the main causes of blindness worldwide. In 2010, it has been estimated that about 95 million people suffered from a form of DR (Leasher et al., 2016). Due to the increasing number of diabetic people and the increased life expectancy, these numbers are expected to rise in the near future.

DR is a multifactorial progressive disease characterized by an extremely complex pathogenesis involving different factors and a variety of pathophysiologic mechanisms. Hyperglycemia represents a link between diabetes and DR complications. Indeed, prolonged high glucose levels damage the retina, inducing metabolic changes that result in dysregulation of a number of mediators, including growth factors, neurotrophic factors, cytokines/chemokines, vasoactive agents, and inflammatory and adhesion molecules. The altered retinal microenvironment is responsible for the appearance and the progression of extended vascular lesions and cell death (Qian and Ripps, 2011; Ola et al., 2012; Tarr et al., 2013; Abcouwer and Gardner, 2014).

DR has often been regarded as a purely vascular disorder of the retina. Clinically, it is classified as non-proliferative, characterized by microvascular damage, including blood-retina barrier (BRB) breakdown, basement membrane thickening, leukocyte adhesion, occurrence of acellular capillaries, capillary degeneration, pericyte loss; or proliferative, where neoangiogenesis phenomena are observed and new blood vessels are formed. These neovessels may generate a mechanic traction, causing retinal detachment and consequent blindness (Stitt et al., 2013). The key factor involved in pathologic vascular changes, from microvascular damage to neoangiogenesis, is vascular endothelial growth factor (VEGF). Consequently, DR treatments are mainly based on intraocular delivery of anti-VEGF molecules; however, the intravitreal administration of anti-VEGF drugs has several drawbacks, not the least of which is the fact that, due to the short half-life of the drug, frequent intraocular injections are necessary, generating different side effects, such as endophthalmitis and cataracts (Simo et al., 2014; Duh et al., 2017; Zhao and Singh, 2018). In addition, anti-VEGF drugs are used in mid to late stages of DR—when the vascular phenotype becomes evident, the disease is well-established, and vision has been significantly affected. Therefore, new alternative approaches to the current standard are urgently required to develop effective and early treatment options that may counteract the progression of DR at stages preceding the appearance of an evident vessel damage or vessel proliferation.

In addition to, and in contrast with, the view of DR as a purely vascular pathology, several investigations have studied the involvement and the role of retinal neurons in the disease. Indeed, since neurons are the most fragile and demanding cellular elements in the retina, it is conceivable that they are the first to be affected by damage when the microenvironment composition is drastically changed. Consistent with this hypothesis, a large amount of data has been collected in recent years, confirming that considerable damage of retinal neurons is present in early stages of DR (Antonetti et al., 2006; Hernandez and Simo, 2012; Zhang et al., 2013; Jindal, 2015; Simo and Hernandez, 2015; Hernandez et al., 2016b) and that DR may be considered a neurodegenerative disease of the retina (Barber, 2003).

Summarizing the evidence, one can say that both retinal neurons and vessels are affected in DR; therefore, the question is what kind of relationship, if any, exists between neuronal and vascular damage in DR. A first possibility is that there is no relationship and that neurons on one side and vascular elements on the other independently respond to the alterations caused by high glucose. Only at late stages of the disease, when proliferating vessels cause retinal detachment, the vascular pathology would affect neuronal function and survival. This hypothesis seems unlikely because neuronal, glial, and vascular cells are known to be intimately connected in the neurovascular unit, and recently reviewed evidence indicates that glial, neural, and microvascular dysfunctions are interdependent and intimately involved in the development of DR (Hammes, 2018; Simo et al., 2018). In this line, the American Diabetes Association has defined DR as a tissue-specific neurovascular complication involving progressive disruption of the interdependence between multiple cell types in the retina (Solomon et al., 2017; Simo et al., 2018). In particular, the function of the neurovascular unit is precociously affected in DR often before microvascular complications can be appreciated (see Simo and Hernandez, 2015, for references). Therefore, we favor the hypothesis that in DR, retinal neurons are primarily affected and their reaction to stress induces the vascular complications. Supporting this hypothesis, there are observations suggesting that brain damage, together with the activation of death pathways, also stimulates protective mechanisms mediated by chemical signals derived from the injured brain itself (Iadecola and Anrather, 2011). In the case of DR, one of these signals is likely to be represented by VEGF, which would be released by the retina in the early phases of the disease as an immediate response to neuronal stress. Indeed, this growth factor not only is a powerful inducer of vascular responses but is also known to exert important neuroprotective actions in the retina (Azzouz et al., 2004; Saint-Geniez et al., 2008; Romano et al., 2012; Beazley-Long et al., 2013; Foxton et al., 2013; Casini et al., 2014; Hombrebueno et al., 2015; Amato et al., 2016). Consistent with this view, glutamate excitotoxicity, one of the major causes of retinal neuronal death in DR, has been reported to upregulate VEGF production in diabetic retinas (Cervantes-Villagrana et al., 2010), while inhibition of NMDA receptors resulted in decreased vitreoretinal VEGF in diabetic rats (Kusari et al., 2010). In general, it is interesting to note that in studies analyzing VEGF in DR models after treatment with neuroprotectants, a decrease in apoptotic markers is often associated with a decrease in VEGF expression and/or release (see for instance Amato et al., 2016, 2018b).

**Figure 1:**
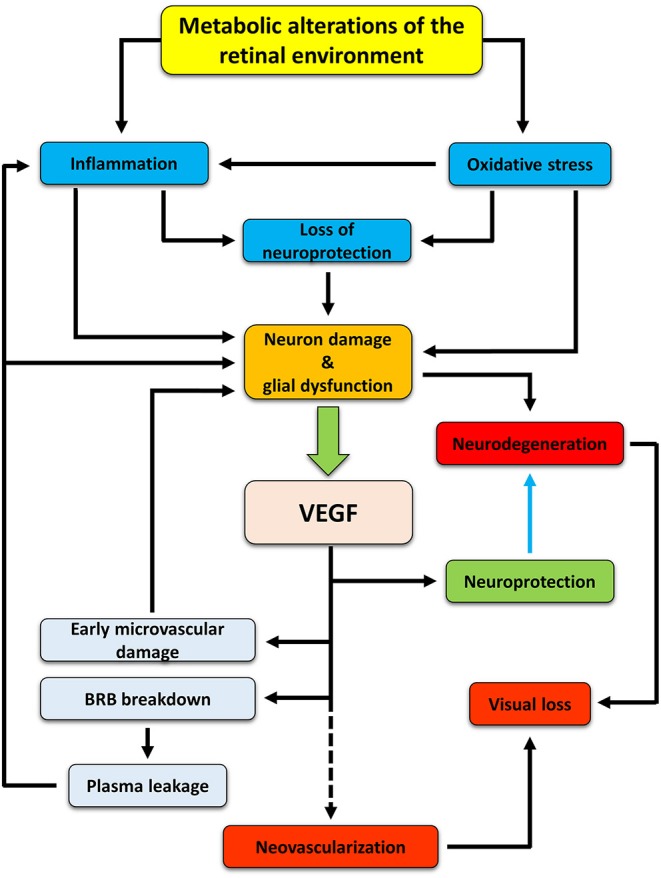
Hypothetic cascade of events occurring in the retina during diabetes and leading to the development of DR. Hyperglycemia induces metabolic changes in the retinal milieu, leading to oxidative stress and inflammation. Oxidative and inflammatory processes cause damages to neuron and glial cells both directly and indirectly by inducing alterations in the production and release of neurotrophic factors. As a consequence, neurodegenerative processes are activated. In an attempt to protect themselves, suffering neurons would trigger production and release of VEGF, mainly by Müller cells, that may act as a neuroprotectant, thus counteracting neurodegeneration (blue arrow). However, if in the early phases of DR VEGF may act as a neuroprotective factor, its prolonged release triggers vascular damages (which, in turn, may reinforce in a different fashion inflammation and retinal damage), ultimately leading to new vessel proliferation. If untreated, neurodegenerative and neovascular processes concur to visual dysfunction, finally leading to vision loss.

In summary, our hypothesis is that, in early DR, VEGF is expressed and released to protect retinal neurons. In this phase, VEGF would not act as a proangiogenic but as a prosurvival factor. Then, a prolonged upregulation of VEGF would lead to microvascular lesions and, further on, to the full manifestation of the pathology (Figure 1). If our hypothesis is correct, then any retinal stress causing neuronal damage should be accompanied by increased VEGF expression and/or release and by vascular changes. Similarly, any compound with neuroprotective properties should also induce VEGF downregulation and amelioration of the vascular lesions. The present review examined a variety of studies in models of DR and of other retinal diseases to highlight the co-occurrence of neuronal damage and VEGF upregulation (with the appearance of vascular lesions) as well as the concomitant neuroprotection and VEGF downregulation (with the amelioration of vascular lesions) in response to neuroprotective treatments.

## Methodology and Definitions

We considered different compounds belonging to different molecular classes but sharing the characteristic of protecting retinal neurons from a variety of stressing conditions. For each compound, a possible correlation between neuroprotective effects and the effects on VEGF expression/release or on vasculopathy has been considered. For the sake of simplicity, only *in vivo* and *ex vivo* studies have been reviewed. A compound was considered “neuroprotective” when it induced a decrease of oxidative stress, inflammation, or apoptotic markers or if it induced an amelioration of retinal function as evaluated, for instance, with electroretinogram (ERG). It was considered “vasoprotective” when it reduced VEGF expression/release, BRB leakage, or vascular lesions (including basement membrane thickening, leukocyte adhesion, occurrence of acellular capillaries, capillary degeneration, pericyte loss). For each compound, papers are first reviewed that documented either neuroprotective or vasoprotective effects of the compound. Then, we considered the papers in which both neuroprotective and vasoprotective effects were documented in the same experimental samples.

## Nutraceuticals

The term “nutraceutical” indicates a food (or part of a food) that can provide health benefits, including the prevention and/or treatment of a disease (Brower, 1998). Nutraceuticals are effective antioxidants since they may act as direct scavengers of reactive oxygen species or they may induce the expression of antioxidant enzymes (Milatovic et al., 2016). They may also exert anti-inflammatory effects by inhibiting pathways linked to the production of inflammatory mediators, including those activated by the nuclear factor kappa-light-chain-enhancer of activated B cells (NF-κB) (Aggarwal et al., 2009). These compounds can be used as natural dietary supplements and therefore can be easily administered, are readily available, and are not likely to induce collateral side effects (Chauhan et al., 2013). Nutraceuticals are known to display neuroprotective effects due to their antioxidant and anti-inflammatory properties and to protect the retina from the vascular damage typical of DR (Rossino and Casini, 2019).

### Curcumin

Curcumin is a yellowish polyphenolic substance constituting the major active compound of *Curcuma longa*. It is largely known for its antioxidant and anti-inflammatory properties (Hewlings and Kalman, 2017), and it may have therapeutic potential for retinal diseases (Wang et al., 2013).

Some studies reported beneficial effects of curcumin on the side of retinal neuroprotection. For instance, in rats with streptozotocin (STZ)-induced diabetes (a model of type 1 diabetes), curcumin inhibited retinal oxidative stress, protected Müller cells, and prevented the downregulation of glutamine synthetase, the enzyme involved in glutamate detoxification and recycling, thus protecting the retinal neurons from glutamate excitotoxicity (Zuo et al., 2013). On the other hand, there are data documenting an inhibitory effect of curcumin on diabetes-induced VEGF upregulation in diabetic rat retinas (Mrudula et al., 2007).

Other studies investigated the neuroprotective actions of curcumin together with its effects on VEGF expression and/or retinal vascular lesions. In particular, in STZ diabetic rats, oral curcumin administrations significantly reduced retinal oxidative stress, inflammation, thinning of the retina, and apoptosis, inhibiting, at the same time, VEGF upregulation and thickening of retinal capillary basement membrane (Kowluru and Kanwar, 2007; Gupta et al., 2011; Yang et al., 2018). Similarly, in a rat model of retinal ischemia-reperfusion, curcumin administered with the food inhibited NF-κB activation, with a consequent decrease of pro-inflammatory cytokines, and protected retinal neurons from apoptosis, while it also reduced the retinal capillary degeneration induced by the ischemic treatment (Wang L. et al., 2011).

### Resveratrol

Resveratrol is a polyphenol found in different plants, such as grapes, peanuts, and berries. Similar to curcumin, it possesses important antioxidant properties (Gerszon et al., 2014).

There are studies reporting neuroprotective effects, while other investigations describe vasoprotective actions of resveratrol in retinal diseases. Indeed, orally administered resveratrol has been reported to decrease oxidative stress, NF-κB activation, and apoptosis in diabetic rat or mouse retinas (Kim et al., 2010; Soufi et al., 2012). On the other hand, additional studies in mice with STZ-induced diabetes documented the efficacy of resveratrol in decreasing diabetes-induced retinal VEGF upregulation, pericyte loss, and BRB breakdown (Kim et al., 2012).

Different studies have reported concomitant protective effects of resveratrol against diabetes-induced retinal inflammation or apoptosis of retinal cells on one side and VEGF overexpression, BRB leakage, or leukocyte adhesion on the other (Kubota et al., 2011; Sohn et al., 2016; Chen Y. et al., 2019). Similarly, in a mouse model of endotoxin-induced uveitis, resveratrol led to significant and dose-dependent suppression of oxidative stress, NF-κB activation, and leukocyte adhesion (Kubota et al., 2009).

### Carotenoids

The carotenoids lutein and zeaxantin are the main constituents of oranges, yellow fruits, and dark green leafy vegetables. Together with meso-zeaxanthin, they form the macular pigment of primate eyes and prevent oxidative damage to the retina (Jia et al., 2017).

Likely due to its antioxidant properties, lutein is a recognized protective agent in the retina. In particular, in models of DR or of light-induced retinal degeneration, lutein was reported to preserve neurotrophin levels, protect retinal cells from apoptosis, and prevent both the oxidative stress and functional visual impairment caused by the disease (Sasaki et al., 2010, 2012; Hu et al., 2012; Ozawa et al., 2012).

Several papers have reported an effect of lutein and zeaxantin favoring both retinal cell protection and retinal function on one hand and inhibition of VEGF increase and vascular lesions on the other. Indeed, in retinas of STZ rats, zeaxantin inhibited the diabetes-induced oxidative stress as well as the upregulation of VEGF and intercellular adhesion molecule-1 (ICAM-1), an indicator of leukocyte adhesion (Kowluru et al., 2008). In addition, in the rat STZ model, a nutritional supplement containing lutein, zeaxantin, and other nutrients preserved retinal function, as evaluated with ERG, and at the same time reduced the diabetes-induced increase of NF-κB activation and interleukin-1β (IL-1β) expression, while it decreased VEGF and capillary degeneration (Kowluru et al., 2014). Similarly, in an obesity-induced high-fat diet rat model, lutein and zeaxantin, or meso-zeaxantin, reduced oxidative stress by promoting the expression of antioxidant enzymes and inhibited NF-κB activation, while they also inhibited VEGF and ICAM-1 upregulation and vascular pathology (Orhan et al., 2016; Tuzcu et al., 2017).

### Catechins

Green tea is a popular beverage rich in catechin, epicatechin, epigallocatechin, epicatechin gallate, and epigallocatechin gallate. Among these, epigallocatechin gallate is the most abundant catechin in green tea and possesses antioxidant and anti-inflammatory activities (Chu et al., 2017).

Catechins have been shown to exert powerful anti-inflammatory effects in the retinas of STZ rats by decreasing NF-κB activation and the production of inflammatory factors, such as tumor necrosis factor α (TNFα), IL-6, and IL-1β (Wang N. et al., 2018). In addition, epicatechin has been shown to exert neuroprotective effects in retinas of diabetic rats likely by reducing the production of the precursor form of nerve growth factor (Al-Gayyar et al., 2011). On the vascular side, recent observations reported an effect of epigallocatechin-3-gallate in reducing vascular leakage and permeability in an *in vivo* model of VEGF-induced BRB breakdown (Lee et al., 2014).

There is evidence of concomitant neuroprotective and vasoprotective effects of catechins in rat models of DR. In particular, orally administered green tea was observed to protect the diabetic retina against oxidative stress and promote glutamate uptake by Müller cells. It also preserved retina functionality, as demonstrated by ERG responses, and reduced BRB permeability, as demonstrated by reduced downregulation of occludin, a tight junction protein of the BRB (Silva et al., 2013). In addition, green tea was observed to prevent not only the diabetes-induced decrease of antioxidant enzymes and the increase of TNFα but also VEGF upregulation and the increase of retinal capillary basement membrane thickness (Kumar et al., 2012a).

### Hesperetin

Hesperetin is a flavonoid polyphenol that is commonly present in citrus fruits and has been reported to exert antioxidant effects in diabetic retinas (Rossino and Casini, 2019).

In rodent models of retinal ischemia-reperfusion, hesperetin displayed potent neuroprotective actions as it prevented oxidative stress and apoptosis and preserved retinal layer thickness (Kara et al., 2014; Shimouchi et al., 2016). In addition, in retinas of STZ-treated rats, hesperetin administrations significantly reduced VEGF overexpression, BRB leakage, and pathologic vascular changes (Kumar et al., 2012b).

There is only one report in the literature concerning neuroprotective and vasoprotective effects of hesperetin in the same experimental material. In retinas of STZ-induced diabetic rats, orally administered hesperetin was effective in promoting antioxidant enzyme expression and preventing the increase of the pro-inflammatory cytokines TNFα and IL-1β and of apoptotic markers, while a protective effect of limiting the increase of basement membrane thickness was also reported (Kumar et al., 2013).

### Other Nutraceuticals

There are a number of additional nutraceuticals that have been sporadically reported to exert both neuroprotective and vasoprotective effects, mostly in models of DR.

Quercetin, a common flavonoid polyphenol found in vegetables and fruits, has been reported to protect the diabetic retina from oxidative stress, inflammation, and histopathologic changes (Kumar et al., 2014; Ola et al., 2017) probably by promoting the expression of neurotrophic factors (Ola et al., 2017). Notably, quercetin has also been reported to prevent diabetes-induced retinal VEGF upregulation (Chen B. et al., 2017). Chrysin, another natural flavonoid, is found in herbs and honeycomb. It may exert neuroprotective effects since it has been shown recently to protect retinal photoreceptors by maintaining valid retinoid visual cycle-related components in the retinal pigment epithelium of diabetic rats (Kang et al., 2018). It has also been observed to inhibit VEGF upregulation, BRB leakage, and vascular lesions in the retinas of diabetic *db/db* mice (Kang et al., 2016). Anthocyanins constitute a further class of flavonoids, which are responsible for the red or blue color of plants, fruits, and flowers. Blueberry anthocyanins have been observed to protect diabetic rat retinas from oxidative stress and decrease VEGF levels in these same retinas (Song et al., 2016), while a *Vaccinium myrtillus* extract, containing large amounts of anthocyanins, reduced VEGF expression and preserved BRB integrity in retinas of STZ rats (Kim et al., 2015).

Different compounds have been described to exert at the same time neuroprotective and vasoprotective effects. For instance, eriodictyol, one of the most abundant dietary flavonoids, administered to STZ rats inhibited the retinal expression of the pro-inflammatory cytokine TNFα, while it also decreased the retinal levels of VEGF and of ICAM-1 and suppressed BRB breakdown (Bucolo et al., 2012). In both an *ex vivo* mouse model of retinal oxidative stress and the *in vivo* STZ rat model, Lisosan G, a fermented powder obtained from organic whole grains, has been described recently to exert powerful antioxidant, antiapoptotic, and anti-inflammatory actions. It also preserved retinal function, as evaluated with ERG. Concurrently, it inhibited upregulation of retinal VEGF and prevented BRB breakdown (Amato et al., 2018b). Similarly, an ethanolic extract of *Morus alba* leaves displaying high free radical scavenging activity reduced oxidative stress, inflammation, apoptosis, and VEGF expression in retinas of STZ rats (Mahmoud et al., 2017). Also, the traditional Chinese prescription *Tang Wang Ming Mu* granule has been found to protect diabetic rat retinas from oxidative stress and inflammation reducing at the same time retinal VEGF levels and vascular changes (Chen M. et al., 2017). Finally, kaempferol, a flavonol found in tea, broccoli, apples, strawberries, and beans, protected rat retinas from sodium iodate-induced retinal degeneration by reducing histopathologic changes and apoptosis, while it also reduced the upregulated VEGF protein expression (Du et al., 2018).

Taken together, these studies with nutraceuticals documented an action of these compounds that was at the same time both neuroprotective and vasoprotective. Indeed, the data, prevalently obtained in rodent models of DR, revealed that nutraceuticals, acting as antioxidant and/or as anti-inflammatory agents, not only are effective in reducing retinal neurodegeneration but also prevent the deleterious increase of VEGF levels and consequent vascular lesions.

## Antioxidants

The nutraceuticals discussed above display important antioxidant capabilities, but other antioxidant compounds have been also described, which may act in retinal diseases and protect both retinal neurons and vessels.

### Calcium Dobesilate

Calcium dobesilate (CaD) is an oxygen free radical scavenger (Brunet et al., 1998; Szabo et al., 2001). It is considered a vasoprotective drug, and it has been approved for the treatment of DR in several countries for many years (Tejerina and Ruiz, 1998; Berthet et al., 1999); however, it has not been widely used in clinical practice. In effect, CaD exerts multifaceted actions contrasting neurovascular unit impairment, and therefore, it can be considered a good candidate drug for targeting the early stages of DR. In particular, the effects of CaD in DR have been recently reviewed, and they include (i) reduction of capillary permeability and consequent BRB leakage; (ii) inhibition of endothelial cell apoptosis; (iii) antioxidant activity and protection against reactive oxygen species; and (iv) inhibition of the expression of VEGF and ICAM-1 (Zhang et al., 2015).

In retinas of *db/db* mice (a model of type 2 diabetes) and in retinas of STZ rats, CaD significantly reduced biomarkers of oxidative stress and NF-κB activation with consequent decrease of pro-inflammatory cytokines, such as TNFα, IL-1β, IL-6, and IL-8 (Bogdanov et al., 2017; Voabil et al., 2017). In addition, in STZ rats, CaD reduced vascular leakage and VEGF expression (Rota et al., 2004). Both neuroprotective and vasoprotective effects of CaD have been described in diabetic retinas. Indeed, in retinas of STZ rats, in addition to protective effects against oxidative stress, inflammation, and retinal thinning, CaD has been reported to exert inhibitory effects against diabetes-induced BRB breakdown, downregulation of tight junction protein expression, increased VEGF and ICAM-1 expression, and leukocyte adhesion (Leal et al., 2010). In retinas of *db/db* diabetic mice, CaD significantly decreased diabetes-induced oxidative stress and retinal cell apoptosis. In addition, it reduced glutamate extracellular concentration, by preventing glutamate transporter downregulation, and improved ERG responses. At the same time, CaD inhibited VEGF upregulation and vascular leakage (Sola-Adell et al., 2017).

### Other Antioxidants

It is clear that suppression of antioxidant defenses is deleterious to the retina. For this reason, recent studies have focused on the ability of some antioxidant compounds to regulate antioxidant gene expression, such as the nuclear factor erythroid-2-related factor 2 (Nrf2) activator dh404 and a DNA methyltransferase (DNMT) inhibitor.

Nrf2 is a redox-sensitive transcription factor that is kept in a latent state until an increase in free radical concentration releases Nrf2, which enters the cell nucleus and initiates the transcription of antioxidant genes (Di Marco et al., 2015). Boosting Nrf2 with a specific activator increases the transcription of antioxidant genes and therefore may protect tissues from oxidative damage. In retinas of STZ rats, the Nrf2 activator dh404 has been reported not only to decrease oxidative stress and the expression of inflammatory mediators but also to prevent VEGF upregulation and vascular leakage (Deliyanti et al., 2018).

It has been observed that DNA methylation may be involved in the regulation of gene expression in the retina during the progression of DR (Kowluru et al., 2015; Mishra and Kowluru, 2016, 2019). In particular, DNMT inhibitors may favor the expression of antioxidant genes. Indeed, in diabetic rat retinas, DNMT inhibition restored antioxidant enzyme expression and, in parallel, also prevented the diabetes-induced increase of VEGF and of ICAM-1 expression (Xie et al., 2019).

These observations on the effects of antioxidant compounds in models of DR indicate that reduction of oxidative stress is accompanied by positive effects on the vascular pathology and therefore favors both neuroprotective and vasoprotective actions.

## Neuropeptides

Neuropeptides are short to medium amino acid chains, which function primarily as complementary signals to “classic” neurotransmitters to fine-tune neurotransmission (Hokfelt et al., 2003). Some of them have been found to be important for the regulation of cell death/survival in different neuronal systems, where they express important neuroprotective properties (Catalani et al., 2017; Reglodi et al., 2017; Chen X.Y. et al., 2019). Neuropeptides and their receptors are widely expressed in mammalian retinas, where they exert multifaceted functions both during development and in the mature animal (Bagnoli et al., 2003). In particular, some of them may exert important roles in retinal diseases (Gabriel, 2013; Cervia et al., 2019).

### Glucagon-Like Peptide-1

Glucagon-like peptide-1 (GLP-1) is known as a hormone secreted by the gastrointestinal tract in response to food, stimulating insulin and inhibiting glucagon secretion (Drucker and Nauck, 2006). GLP-1 has also been recognized as a neuropeptide. Indeed, GLP-1 and its receptor GLP-1R are expressed in the brain, where they influence multiple neural circuits modulating feeding behavior and reward (Smith et al., 2019). Both GLP-1 and GLP-1R are expressed in mammalian retinas (Zhang et al., 2009; Zhang Y. et al., 2011; Hernandez et al., 2016a; Cai et al., 2017; Hebsgaard et al., 2018).

Neuroprotective effects of GLP-1R activation have been demonstrated in a rat model of optic nerve crush, where intravitreal implants of beads with genetically modified cells producing GLP-1 decreased apoptosis and promoted survival of retinal ganglion cells (Zhang R. et al., 2011), and in diabetic rats, where exendin-4, an analog of GLP-1, protected from oxidative stress from apoptotic cell death and ameliorated retinal function as assessed with ERG (Zhang et al., 2009; Zhang Y. et al., 2011; Fan et al., 2014b; Zeng et al., 2016; Cai et al., 2017; Cervia et al., 2019). Most importantly, both neuroprotective and vasoprotective effects of GLP-1 agonists have been described in models of retinal diseases. For instance, in a rat model of retinal ischemia-reperfusion, exendin-4 suppressed inflammatory gene expression and reduced BRB permeability (Goncalves et al., 2016). Strong evidence for a double action of GLP-1 as a neuroprotectant and vasoprotectant also comes from studies in models of DR. Indeed, recent studies in rodent retinas have reported that GLP-1 or GLP-1R agonists may exert a neuroprotective action since they improved retinal function, as assessed with ERG, protected retinal cells from death, reduced oxidative stress and IL-1β expression, and inhibited the increase of extracellular glutamate. At the same time, these compounds induced vasoprotection since they decreased VEGF levels, preserved the expression of tight junction proteins of the BRB, reduced BRB leakage, and inhibited the increase of ICAM-1 levels (Fan et al., 2014a; Hernandez et al., 2016a; Sampedro et al., 2019).

Similar to GLP-1R agonists, inhibitors of dipeptidyl peptidase 4 (DPP4, the GLP-1 degrading enzyme) have been tested for their potential use in DR treatments. The data of different studies indicated that DPP4 inhibitors, such as linagliptin, saxagliptin, or sitagliptin, efficiently increase retinal GLP-1 levels and that this increase, in rodent models of DR, is correlated with reduced oxidative stress, inflammation (as assessed by IL-1β levels), extracellular glutamate levels and neuronal apoptosis and with preservation of retinal function. At the same time, DPP4 inhibitors induced amelioration of different vascular features, including diabetes-induced changes in the subcellular distribution of the tight junction proteins occludin, claudin-5, and zonula occludens-1; BRB breakdown; ICAM-1 upregulation; pericyte loss; and formation of acellular capillaries (Goncalves et al., 2012, 2014; Dietrich et al., 2016; Hernandez et al., 2017).

### Somatostatin

Somatostatin (somatotropin release inhibiting factor, SRIF) is expressed in the retina, together with its five receptor subtypes (named sst1-5), where they express important physiological functions (Casini et al., 2005; Cervia et al., 2008a). Low vitreous levels and low intraocular production of SRIF have been found in patients with diabetic macular edema, chronic uveitis macular edema, and quiescent intraocular inflammation (Simo et al., 2007; Fonollosa et al., 2012), suggesting that SRIF alterations may be directly involved in the pathogenesis of these conditions. In addition, a variety of experimental observations suggested that SRIF may exert powerful neuroprotective effects in different retinal diseases (Cervia et al., 2008a; Cervia and Casini, 2013; Hernandez et al., 2014; Wang et al., 2017).

The SRIF analog pasireotide and SRIF receptor agonists targeting the sst2 or sst5 receptors were found to significantly protect rat retinal neurons in *in vivo* models of AMPA excitotoxicity (Kiagiadaki and Thermos, 2008; Kiagiadaki et al., 2010; Kokona et al., 2012). In addition, in a retinal ischemia-reperfusion mouse model, SRIF mediated the neuroprotective and anti-inflammatory effects of capsaicin, a selective agonist for transient receptor potential vanilloid type-1, a ligand-gated non-selective cation channel (Wang et al., 2017). Moreover, in retinas of STZ rats, topical SRIF administrations prevented glutamate accumulation, apoptosis, and ERG abnormalities (Hernandez et al., 2013). Similarly, studies in *ex vivo* ischemic retinas of mice or rats reported that SRIF, its analogs, or the constitutive activation of the sst2 receptor significantly preserved retinal neurons from ischemia-induced morphological changes and apoptosis (Catalani et al., 2007; Cervia et al., 2008b; Kokona et al., 2012). Moreover, in retinal explants in which hypoxic conditions induced the expression of apoptotic markers, the sst2-preferring SRIF analog octreotide reduced apoptotic signals (Dal Monte et al., 2012). Finally, in *ex vivo* explants of mouse retinas treated with high glucose, octreotide prevented apoptosis of retinal neurons, likely stimulating an increase of the autophagic flux (Amato et al., 2018a). Interestingly, a study in *ex vivo* ischemic mouse retinas reported that acute ischemia induces a sudden increase in VEGF release from neurons, suggesting that VEGF may represent a stress signal released by retinal neurons when their integrity is threatened. Supporting this view, the neuroprotective SRIF analog octreotide reduced VEGF release from ischemic retinas (Cervia et al., 2012).

Some studies reported concomitant effects of SRIF, or its analogs, on neuroprotection and vasoprotection. For instance, in a mouse model of retinal ischemia-reperfusion, octreotide has been reported to protect from oxidative stress, inflammation (as assessed by NF-κB activation), and neuronal death, while it also significantly reduced ICAM-1 expression, indicating decreased leukocyte adhesion (Wang et al., 2015). In *ex vivo* ischemic mouse retinas, octreotide inhibited the ischemia-induced increase of oxidative stress, glutamate levels, apoptosis, and VEGF expression (D'alessandro et al., 2014). Similar observations were reported in *ex vivo* mouse retinal explants challenged with high glucose, oxidative stress, or advanced glycation end products (AGE), toxic products that accumulate under hyperglycemic conditions and that are likely to play an important role in the pathogenesis of DR. In particular, these studies showed that protecting retinal neurons from diabetic stress also reduces VEGF expression and release, while inhibiting VEGF leads to exacerbation of apoptosis (Amato et al., 2016). Therefore, the retina in early DR may release VEGF as a prosurvival factor, and a neuroprotective agent such as octreotide may decrease the need of VEGF production by the retina, therefore limiting the vasculopathy associated with VEGF upregulation.

### Angiotensin

The renin-angiotensin system (RAS) is involved in the regulation of blood pressure. Angiotensin I (AngI) is generated from the proteolytic cleavage of angiotensinogen, a reaction catalyzed by the enzyme renin. AngI is further processed by angiotensin-converting enzyme (ACE) and ACE2 to angiotensin II (AngII), the main effector of the RAS, acting at the angiotensin type 1 and type 2 receptors (AT1R and AT2R) (Fletcher et al., 2010). A local RAS is present in the retina, where RAS components have been localized to different retinal cell types, including retinal neurons and Müller cells (Wilkinson-Berka et al., 2012). A variety of studies have shown that reduction of AngII expression or blockade of AT1R on the one hand, or stimulation of ACE2 on the other, may reduce the retinal damage occurring in retinal pathologies, such as glaucoma, retinal ischemia, autoimmune uveitis, or DR (Cervia et al., 2019).

Several investigations have provided evidence for a neuroprotective role exerted by AT1R inhibitors in different models of retinal diseases. For instance, inhibitors like valsartan, losartan, or candesartan were effective in attenuating light-induced retinal damage in mice by reducing oxidative stress and improving ERG responses (Narimatsu et al., 2014). Similarly, candesartan prevented ganglion cell loss, thinning of the retina, and ERG deficits in a retinal excitotoxicity mouse model (Semba et al., 2014). In mice with increased intraocular pressure, used as models of glaucoma or ischemia-reperfusion, AT1R blockade reduced oxidative stress, inhibited the increase of extracellular glutamate, and mitigated ganglion cell loss (Yang et al., 2009; Fujita et al., 2012; Liu et al., 2012; Quigley et al., 2015). In rats or mice with STZ-induced diabetes, blockers of AT1R, in addition to protecting the retina from oxidative stress, apoptotic cell death, and histopathologic damage (Silva et al., 2009; Ola et al., 2013; Thangaraju et al., 2014), also preserved mitochondrial integrity, increased the expression of neurotrophic factors, and improved functional ERG responses (Silva et al., 2009; Ozawa et al., 2011; Ola et al., 2013). In experimental models of mouse autoimmune uveitis or endotoxin-induced uveitis, the delivery of different formulations of ACE2 and/or its product Ang(1-7) or the administration of an ACE2 activator reduced retinal inflammation (Qiu et al., 2014, 2016; Shil et al., 2014) and prevented histologic damage as well as ERG abnormalities (Qiu et al., 2016). Similarly, both in an experimental glaucoma model and in STZ rats, retinal ganglion cells were protected from apoptotic cell death by the administration of an ACE2 activator (Foureaux et al., 2013, 2015). On the vascular side, in retinas of STZ rats evidence was provided of an inhibitory effect on VEGF expression and on leukocyte adhesion exerted by a prorenin receptor blocker (Satofuka et al., 2009).

There are a few studies documenting concomitant effects of the Ang system both on the side of neuroprotection and on that of vasoprotection. One study reported that in diabetic rats, increased plasma prorenin levels exacerbated the expression of inflammatory cytokines, retinal apoptotic cell death, as well as the formation of acellular capillaries, while a prorenin receptor blocker significantly reduced these effects (Batenburg et al., 2014). Similarly, adeno-associated virus-mediated gene delivery of ACE2 or Ang(1-7) significantly reduced diabetes-induced oxidative damage, inflammation, retinal vascular leakage, and formation of acellular capillaries in both diabetic mice and rats (Verma et al., 2012).

These data support the possibility that the documented neuroprotective actions of AngII blockade or ACE2 stimulation also influence the vascular pathology and induce an amelioration of the vascular traits (VEGF upregulation, acellular capillaries, leukocyte adhesion, BRB breakdown) especially in models of DR.

### Pituitary Adenylate Cyclase-Activating Polypeptide and Vasoactive Intestinal Peptide

Pituitary adenylate cyclase-activating polypeptide (PACAP) and vasoactive intestinal peptide (VIP) belong to the same peptide superfamily, which also includes secretin and glucagon. The PACAP receptors can be classified into two groups: PACAP receptor 1 (PAC1), which binds PACAP with higher affinity than VIP, and vasoactive intestinal polypeptide receptors (VPAC1 and VPAC2), which bind PACAP and VIP with similar affinities (Vaudry et al., 2009).

PACAP and PAC1R have been detected in the retina, where they are involved in neurotransmission, neuromodulation, and, mostly, neuroprotective functions (Nakamachi et al., 2012). VPAC2 expression has also been reported in the mouse retina (Harmar et al., 2004). The retinoprotective effects of PACAP have been the subject of a variety of studies, and these data have been excellently reviewed (Atlasz et al., 2010; Nakamachi et al., 2012; Shioda et al., 2016). Further evidence has been provided by more recent studies. Indeed, PACAP has been shown to inhibit apoptosis and promote survival of retinal ganglion cells in different models of retinal injury (Lakk et al., 2015; Ye et al., 2019). In addition, intravitreal or topical administrations of PACAP or a PAC1 agonist to ischemic retinas *in vivo* ameliorated ERG responses, prevented inflammation, and reduced the thinning of retinal layers and the loss of cells in the ganglion cell layer (Danyadi et al., 2014; Vaczy et al., 2016; Werling et al., 2017; Atlasz et al., 2018). Similarly, PACAP intraocular delivery in rats with STZ-induced diabetes protected the retina from apoptosis and maintained retinal synaptic integrity (Szabadfi et al., 2014, 2016). PACAP was also demonstrated to contrast the diabetes-induced modifications of the expression of hypoxia-inducible factors (HIFs), among which HIF-1 is the main regulator of VEGF expression (D'amico et al., 2015).

Both neuroprotective and vasoprotective effects of PACAP have been documented in retinas of STZ rats and in ischemic retinas *in vivo*, where PACAP reduced thinning of retinal layers and prevented the expression of both inflammatory cytokines and VEGF (Werling et al., 2016; D'amico et al., 2017b). In addition, in an *ex vivo* model of retinal ischemia, PACAP effectively decreased oxidative stress, glutamate accumulation, inflammatory mediators, and apoptosis. At the same time, it also decreased VEGF expression, which was upregulated in the ischemic retina (D'alessandro et al., 2014). Finally, in *ex vivo* retinal explants stressed with high glucose, oxidative stress, or AGE, the strong PACAP antiapoptotic effects were paralleled by inhibition of the stress-induced increase of VEGF expression and release (Amato et al., 2016).

VIP is expressed in the retina in a population of amacrine cells (Perez De Sevilla Muller et al., 2019). It has been reported to reduce retinal neurodegeneration caused by ischemia-reperfusion injury, promoting an antioxidant effect (Tuncel et al., 1996). The neuroprotective effects of VIP may be mediated by activity-dependent neurotrophic protein (ADNP) (Bassan et al., 1999; Zusev and Gozes, 2004; Giladi et al., 2007). Indeed, both ADNP and an 8-amino acid peptide derived from ADNP (referred to as NAP) display important neuroprotective activities (Magen and Gozes, 2014). Interestingly, NAP seems to exert protective effects against both the neural and the vascular pathology induced by DR, as it reduced inflammation (D'amico et al., 2019) and apoptosis (Scuderi et al., 2014) as well as the levels of the α subunit of HIF-1 (HIF-1α) and VEGF in retinas of rats with STZ-induced diabetes (D'amico et al., 2017a).

### Other Peptides

α-Melanocyte-stimulating hormone (α-MSH) is a widely-distributed 13-amino acid peptide derived from proteolytic cleavage of proopiomelanocortin (Wardlaw, 2011). It acts at five subtypes of G protein-coupled receptors designated MC1R to MC5R (Yang, 2011). α-MSH protected the rat retina from both functional and structural damage induced by ischemia-reperfusion (Varga et al., 2013), suppressed inflammation and maintained retinal structure in a mouse model of experimental autoimmune uveitis (Edling et al., 2011), and protected photoreceptors from degeneration in a rat model of retinal dystrophy (Naveh, 2003). In a rat model of STZ-induced diabetes, intravitreal injections of α-MSH reduced oxidative stress, inflammation, and apoptosis, while they also inhibited the expression of ICAM-1 (Zhang et al., 2014), indicating reduced leukostasis. In early diabetic retinas, α-MSH also reduced inflammation, ameliorated ERG responses, and reduced retinal thinning, while it inhibited BRB breakdown and vascular leakage, likely acting at MC4R (Cai et al., 2018).

Endothelin (ET) is a potent vasoconstrictor composed of three isoforms designated ET-1, ET-2, and ET-3, whose actions are mediated by the ET type A receptor (ETRA) and ET type B receptor (ETRB) (Davenport et al., 2016). There are indications that ET activity may be involved in DR, and evidence has been provided that ET inhibition may ameliorate the pathologic signs of DR. Indeed, an ETRA antagonist has been reported to block the diabetes-induced upregulation of both VEGF and ICAM-1 in retinas of STZ rats (Masuzawa et al., 2006), while other observations have described positive effects of ETR inhibition on both neuronal and vascular changes seen in DR. For instance, ETRA and/or ETRB inhibitors reduced retinal thinning, the number of apoptotic cells, and the levels of the pro-inflammatory cytokine TNFα in diabetic rat retinas, and at the same time they also reduced pericyte loss, capillary degeneration, vascular leakage, and the levels of both VEGF and ICAM-1 (Chou et al., 2014; Alrashdi et al., 2018; Bogdanov et al., 2018).

Erythropoietin (EPO) stimulates erythroid progenitor cell and early erythroblast maturation and is mainly used in anemia treatment (Jelkmann, 2013). EPO is expressed in the retina (Hernandez et al., 2006; Fu et al., 2008), where it exerts well-documented neuroprotective functions (Kilic et al., 2005; Chung et al., 2009; Colella et al., 2011; Chang et al., 2013). Both neuroprotective and vasoprotective actions of EPO have been described. Indeed, in retinas of STZ rats, EPO was reported to significantly decrease oxidative stress, apoptotic neurodegeneration, and retinal thinning on one hand, and VEGF upregulation, BRB breakdown, and pericyte loss on the other (Zhang et al., 2008; Wang et al., 2010; Wang Q. et al., 2011). Similarly, carbamylated erythropoietin, an EPO derivative, protected diabetic rat retinas from retinal thinning, neuron apoptosis, and functional deficits as evaluated with ERG, while it also reduced VEGF upregulation and vascular leakage (Liu et al., 2015).

In addition to the peptides cited above, some evidence exists for an effect of a few other peptides that is both neuroprotective and vasoprotective. Indeed, antioxidative, anti-inflammatory, and/or antiapoptotic actions together with effects preventing VEGF upregulation and/or BRB breakdown have been described for the peptides growth hormone-releasing hormone (Thounaojam et al., 2017), insulin (Rong et al., 2018), melatonin (Djordjevic et al., 2018), substance P (D'alessandro et al., 2014), and vasoinhibins (Garcia et al., 2008; Arredondo Zamarripa et al., 2014).

Together, these data demonstrate that, similar to the nutraceuticals and antioxidants reviewed above, the powerful neuroprotective effects exerted by different classes of neuropeptides also result in VEGF downregulation and attenuation of the vascular damage in various models of retinal disease.

## Other Factors

The urokinase-type plasminogen activator (uPA) receptor (uPAR) is a glycosylphosphatidylinositol-anchored receptor activated by uPA. uPAR lacks a transmembrane domain, however, it can activate intracellular signaling pathways through lateral interactions with other cell surface receptors, including integrins, G-protein–coupled receptors, and receptor tyrosine kinases, thus forming a system that is involved in many pathological processes, including retinal diseases (Cammalleri et al., 2019b). Recently, the inhibition of the uPAR system has been found to be effective in slowing down cone degeneration and visual dysfunction in a mouse model of retinitis pigmentosa (Cammalleri et al., 2019a). Of interest for this review, in two different models of DR, the STZ rat model mimicking type 1 diabetes (Navaratna et al., 2008; Cammalleri et al., 2017b) and the Torii rat model mimicking type 2 diabetes (Cammalleri et al., 2017a), inhibiting the uPAR system not only ameliorated diabetes-induced ERG dysfunction and reduced inflammation and apoptosis but also resulted in inhibition of VEGF upregulation and BRB breakdown.

Brimonidine is an α2 adrenergic agonist with extensively documented neuroprotective effects in a variety of models of retinal disease (see for instance Guo et al., 2015; Marangoz et al., 2018). In addition, in retinas of rats with STZ-induced diabetes, it has been reported to induce a significant decrease of VEGF expression and of BRB breakdown to levels similar to those observed in control rats (Kusari et al., 2010). Furthermore, in a mouse model of ischemic optic neuropathy, topically applied brimonidine not only reduced oxidative stress and ganglion cell loss but also decreased HIF-1α and VEGF expression (Goldenberg-Cohen et al., 2009).

Peroxisome proliferator-activated receptor α (PPARα), a hormone-activated nuclear receptor, is known as an important modulator of lipid metabolism (Pyper et al., 2010), which also possesses anti-inflammatory and antioxidant properties (Li et al., 2005; Simo and Hernandez, 2009). The PPARα agonist fenofibrate has been used clinically as a triglyceride-lowering drug. However, it seems that downregulation of PPARα in the retina plays a major role in the pathogenesis of DR (Hu et al., 2013), and two independent perspective clinical trials demonstrated that fenofibrate had unprecedented therapeutic effects in DR (Keech et al., 2007; Chew et al., 2010). Emerging evidence suggests that fenofibrate exerts a broad range of beneficial effects on diabetic complications acting against oxidative stress, inflammation, cell death, and angiogenesis (Noonan et al., 2013). In particular, in rodent models of DR, different studies documented the protective effects of fenofibrate or another PPARα agonist against oxidative stress, inflammation, retinal cell death, and decreased retinal function on one hand, and VEGF upregulation, vascular leakage, thickening of capillary basement membrane, ICAM-1 expression, and leukostasis on the other (Chen et al., 2013; Deng et al., 2017; Li et al., 2018; Liu et al., 2018, 2019; Wang N. et al., 2018; Qiu et al., 2019).

Acetaldehyde dehydrogenase 2 is a rate-limiting enzyme for alcohol metabolism, which has been shown to exert neuroprotective effects (Deza-Ponzio et al., 2018). In retinas of STZ rats, it has been reported to promote antioxidant enzyme activity, reduce the expression of proinflammatory cytokines, ameliorate ERG, and significantly reduce VEGF expression (He et al., 2018).

The last example, in this review, of a neuroprotective factor that also ameliorates vascular changes in retinal disease is not concerned with a compound but involves a procedure. Indeed, it is known that ischemic conditioning can be considered a form of protection against ischemic injury through the initiation of endogenous protective mechanisms (Heusch, 2013; Li et al., 2017). As expected, in retinas of STZ rats, ischemic conditioning produced anti-inflammatory and antioxidant effects, and it also protected ganglion cells from death. Interestingly, diabetic retinas treated with ischemic conditioning also showed a significantly downregulated VEGF protein expression (Ren et al., 2018).

## Conclusion

The experimental data summarized in this review of the literature clearly indicate that, in a variety of experimental models of retinal disease, a neuroprotective treatment is efficacious in preventing the vascular changes that are usually associated with the disease (see Table 1 for a comprehensive summary of the data). Although the participation of VEGF is crucial in the early stages of DR, we cannot exclude the participation of some other important molecules, such as the neuronal guidance cues, including ephrins, netrin, and semaphorins, which are also released early by damaged neurons and may promote or attenuate the development of DR (Moran et al., 2016). These molecules are highly expressed in the retina and vitreous of patients with advanced DR (Umeda et al., 2004; Liu et al., 2011; Cerani et al., 2013; Dejda et al., 2014), and their inhibition, like that of VEGF, could reveal an efficient method to reduce aberrant growth of retinal vessels. The possibility exists that treatments with neuroprotectants, in addition to reducing VEGF expression/release, also limit the release of these molecules, thereby exerting a multitarget effect that results in efficient protection from microvascular damage and subsequent development of advanced stages of DR.

**Table 1:** Summary of the neuroprotective and vasoprotective effects of different compounds in models of retinal disease.

		**Neuroprotection**				**Vasoprotection**			**References**
	***Compound***	OS*	IF*	APO*	FUNC*	VASC*	***BRB***	***VEGF***	
	Curcumin	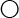	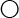						Zuo et al., 2013
								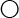	Mrudula et al., 2007
		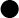	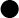					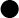	Kowluru and Kanwar, 2007
			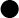	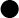		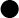			Wang L. et al., 2011
		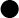	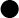					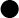	Gupta et al., 2011
		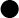		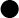		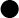		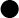	Yang et al., 2018
	Resveratrol			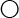					Kim et al., 2010
		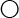	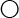	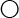					Soufi et al., 2012
						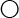	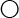	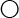	Kim et al., 2012
		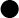	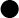			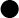			Kubota et al., 2009
			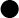			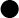		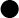	Kubota et al., 2011
			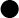				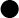		Sohn et al., 2016
			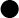	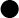			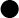	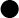	Chen Y. et al., 2019
	Carotenoids	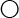		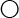					Sasaki et al., 2010
					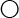				Hu et al., 2012
				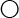	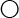				Ozawa et al., 2012
				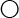					Sasaki et al., 2012
		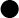				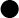		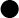	Kowluru et al., 2008
			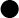		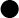	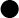		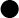	Kowluru et al., 2014
		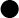	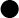			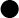		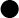	Orhan et al., 2016; Tuzcu et al., 2017
	Catechins			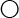					Al-Gayyar et al., 2011
			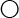						Wang W. et al., 2018
							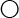		Lee et al., 2014
		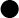	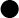			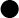		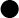	Kumar et al., 2012a
		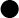			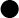		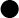		Silva et al., 2013
	Hesperetin			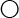					Kara et al., 2014
		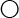		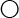					Shimouchi et al., 2016
						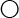	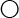	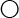	Kumar et al., 2012b
		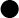	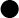	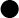		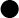			Kumar et al., 2013
	Quercetin	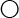	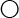	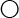					Kumar et al., 2014
				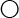					Ola et al., 2017
								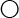	Chen B. et al., 2017
	Chrysin				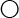				Kang et al., 2018
						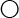	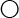	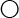	Kang et al., 2016
	Anthocyanins						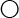	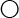	Kim et al., 2015
		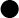						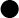	Song et al., 2016
	Eriodictyol		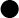			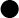	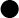	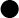	Bucolo et al., 2012
	Lisosan G	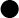	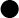	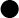	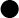		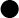	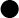	Amato et al., 2018b
	*Morus alba*	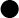	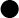	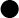				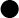	Mahmoud et al., 2017
	*Tang Wang Ming Mu*	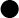	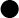			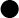		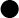	Chen M. et al., 2017
	Kaempferol			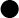				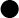	Du et al., 2018
Antioxidants	Calcium dobesilate	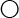	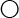						Bogdanov et al., 2017; Voabil et al., 2017
							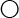	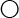	Rota et al., 2004
		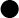	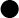	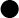		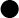	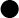		Leal et al., 2010
		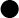		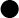	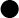		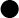	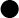	Sola-Adell et al., 2017
	Nrf2 activator	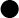	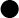				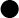	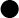	Deliyanti et al., 2018
	DNMT inhibitor	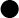				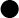		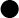	Xie et al., 2019
Neuropeptides	GLP-1			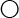	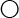				Zhang et al., 2009; Zhang Y. et al., 2011; Fan et al., 2014b
				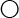					Zhang R. et al., 2011
		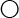		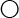	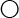				Zeng et al., 2016
		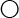		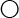					Cai et al., 2017
		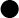	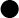	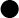			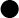		Goncalves et al., 2012
		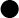	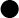		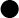	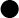	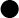	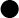	Fan et al., 2014a
			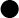	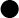			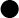		Goncalves et al., 2014
		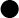	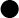	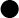		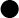			Dietrich et al., 2016
			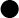				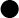		Goncalves et al., 2016
		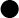	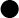	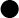	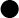	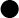	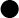	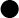	Hernandez et al., 2016a
			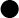	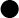	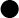		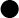		Hernandez et al., 2017
			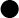	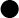	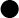		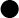	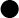	Sampedro et al., 2019
	SRIF			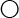					Catalani et al., 2007; Cervia et al., 2008b; Kiagiadaki and Thermos, 2008; Kiagiadaki et al., 2010; Dal Monte et al., 2012; Kokona et al., 2012; Amato et al., 2018a
				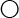	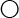				Hernandez et al., 2013
			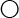	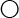					Wang et al., 2017
								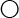	Cervia et al., 2012
		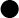		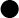				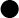	D'alessandro et al., 2014; Amato et al., 2016
		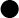	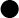	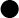		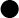			Wang et al., 2015
	Ang	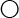		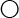					Silva et al., 2009; Fujita et al., 2012; Ola et al., 2013
				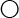					Yang et al., 2009; Liu et al., 2012; Foureaux et al., 2013, 2015; Thangaraju et al., 2014; Quigley et al., 2015
		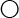		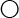	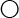				Narimatsu et al., 2014
				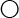	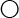				Semba et al., 2014
			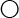						Qiu et al., 2014; Shil et al., 2014
			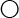	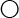	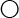				Qiu et al., 2016
						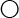		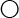	Satofuka et al., 2009
		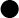	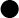			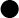	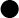		Verma et al., 2012
			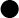	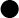		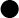			Batenburg et al., 2014
	PACAP				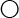				Danyadi et al., 2014
					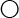				Szabadfi et al., 2014
			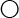	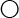					Vaczy et al., 2016
				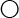					Lakk et al., 2015; Szabadfi et al., 2016; Werling et al., 2017; Atlasz et al., 2018; Ye et al., 2019
		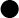		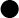				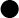	D'alessandro et al., 2014
				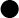				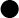	Amato et al., 2016; Werling et al., 2016
			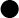					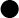	D'amico et al., 2017b
	VIP			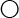					Tuncel et al., 1996
	VIP/NAP			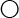					Scuderi et al., 2014
			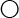						D'amico et al., 2019
								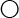	D'amico et al., 2017a
	α-MSH			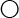					Naveh, 2003
			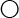	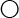					Edling et al., 2011
		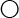		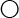	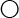				Varga et al., 2013
		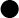	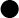	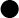		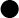			Zhang et al., 2014
			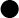	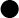	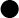	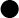	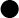		Cai et al., 2018
	ET					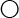		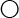	Masuzawa et al., 2006
				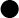		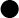			Chou et al., 2014
			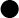				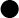	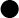	Alrashdi et al., 2018
			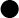	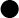				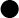	Bogdanov et al., 2018
	Erythropoietin			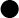			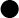		Zhang et al., 2008
		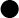				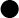			Wang et al., 2010
				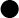		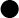		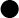	Wang Q. et al., 2011
				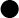	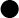		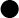	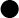	Liu et al., 2015
	GHRH*	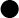	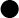	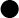			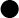	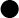	Thounaojam et al., 2017
	Insulin		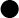	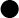			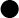	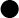	Rong et al., 2018
	Melatonin	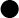						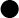	Djordjevic et al., 2018
	Substance P	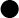		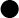				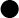	D'alessandro et al., 2014
	Vasoinhibins						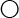		Garcia et al., 2008
		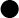					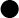		Arredondo Zamarripa et al., 2014
Other factors	uPAR		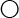	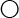	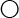				Cammalleri et al., 2019a
							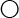		Navaratna et al., 2008
			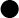	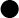	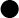		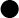	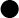	Cammalleri et al., 2017a
			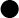		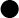		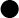	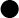	Cammalleri et al., 2017b
	Brimonidine						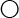	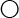	Kusari et al., 2010
				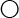					Guo et al., 2015; Marangoz et al., 2018
		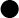		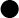				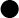	Goldenberg-Cohen et al., 2009
	PPARα agonists		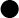				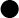	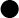	Chen et al., 2013
				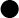	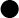	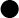	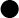		Deng et al., 2017
		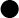	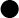			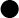	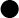	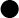	Li et al., 2018
		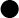	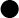			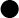	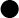		Liu et al., 2018
			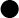	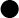				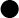	Wang N. et al., 2018
		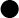				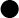			Liu et al., 2019
			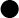		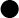	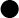		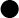	Qiu et al., 2019
	ALDH2*	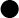	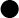		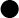			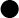	He et al., 2018
	LRIC*	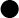	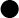	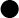				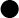	Ren et al., 2018

*The data indicated with “
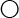
” are from papers documenting either neuroprotective or vasoprotective effects; those indicated with “
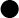
” are from papers documenting both neuroprotective and vasoprotective effects in the same experimental samples. *ALDH2, acetaldehyde dehydrogenase 2; APO, apoptosis; FUNC, retinal function; GHRH, growth hormone-releasing hormone; IF, inflammation; LRIC, remote ischemic conditioning; OS, oxidative stress; VASC, vasculopathy. See “Abbreviations” for the other abbreviations*.

The fact that neuroprotection may limit vascular pathology could be explained assuming that the reviewed compounds may trigger two types of independent, parallel responses: one finalized to neuroprotection and the other affecting the mechanisms regulating VEGF expression and/or release. This might be the case, for instance, for SRIF. Indeed, for the SRIF analog octreotide, there is evidence of an effect, reducing oxidative stress and glutamate release (Dal Monte et al., 2003; D'alessandro et al., 2014), and of a regulatory action on the intracellular mechanisms for VEGF expression (Dal Monte et al., 2009, 2010; Mei et al., 2012). However, most compounds listed in this review are primarily antioxidant and anti-inflammatory substances, which are likely to exert their primary effects on retinal neurons. Therefore, it appears that protecting retinal neurons from stress reduces the probability of VEGF upregulation and the consequent vascular damage. This evidence indicates that treatments with natural substances (nutraceuticals or neuropeptides) during early phases of DR may represent the basis for efficacious therapies of DR that do not impact on the patients' quality of life and that may have only little or no side effects.

## Author Contributions

MR and GC collected the references. MR, MD, and GC organized the layout of the paper and wrote the manuscript.

### Conflict of Interest

The authors declare that the research was conducted in the absence of any commercial or financial relationships that could be construed as a potential conflict of interest.

## Abbreviations

ACE: Angiotensin-converting enzyme
ADNP: Activity-dependent neurotrophic protein
AGE: Advanced glycation end products
AngI: Angiotensin I
AngII: Angiotensin II
AT1R: Angiotensin type 1 receptor
AT2R: Angiotensin type 2 receptor
BRB: Blood-retina barrier
CaD: Calcium dobesilate
DNMT: DNA methyltransferase
DPP4: Dipeptidyl peptidase 4
DR: Diabetic retinopathy
EPO: Erythropoietin
ERG: Electroretinogram
ET: Endothelin
ETRA: ET type A receptor
ETRB: ET type B receptor
GLP-1: Glucagon-like peptide-1
HIF: Hypoxia-inducible factor
HIF-1α: α subunit of HIF-1
ICAM-1: Intercellular adhesion molecule-1
IL-1β: Interleukin-1β
IL-6: Interleukin-6
NF-κB: Nuclear factor kappa-light-chain-enhancer of activated B cells
Nrf2: Nuclear factor erythroid-2-related factor 2
PACAP: Pituitary adenylate cyclase-activating polypeptide
PAC1: PACAP receptor 1
PPARα: Peroxisome proliferator-activated receptor α
RAS: Renin-angiotensin system
SRIF: Somatotropin release inhibiting factor (or somatostatin)
sst: Somatostatin receptor subtype
STZ: Streptozotocin
TNFα: Tumor necrosis factor α
uPA: Urokinase-type plasminogen activator
uPAR: Urokinase-type plasminogen activator receptor
VEGF: Vascular endothelial growth factor
VIP: Vasoactive intestinal peptide
VPAC1: Vasoactive intestinal peptide type 1 receptor
VPAC2: Vasoactive intestinal peptide type 2 receptor
α-MSH: α-Melanocyte-stimulating hormone.
